# Economics of Philanthropy

**Published:** 1898-02-12

**Authors:** 


### The Institutional Workshop.

### ECONOMICS OF PHILANTHROPY.

### I.

"The legislator may think it hard that his power for good is so closely restricted; but he has no reason to complain of any limits upon his power for evil. On the contrary, it would seem that there is no race of men whom a few laws respecting industry, trade, and finance, passed by country squires or labour demagogues, in defiance of economic principles, could not in half a generation transform into beasts."*

This sentence, which is taken from Professor Walker's book on political economy, conveys such a solemn, such a tremendous, warning that it may well give pause, not only to the legislator, but to everyone who desires to help or benefit his fellows. If it is a true saying, then the work of the philanthropist is a more responsible one than he has often realised. To do good is a more difficult thing than the amiable persons who never turn a beggar from their door have any idea of, and, especially in an age which incessantly presses upon the Legislature that it is its duty to do this or that for the poor, it becomes a wise and even a necessary thing to make some inquiry into the economics of philanthropy. By this term we mean an inquiry into the forms which "goodwill towards men" takes, whether these be supported by legislative or voluntary effort, and such a study of their methods and results as will show us in some degree how far they fulfil their purpose. The mission of philanthropy is to relieve poverty; but it is only true philanthropy when it helps to remove the poor man from the necessity of relying on its aid. Like all other forces, it must work either good or evil. The philanthropy which does not elevate tends to pauperise.

We may regard the idea that even charity cannot evade the laws of economic science as a discovery of this century. Yet in the benefactions of mediæval times we find often that the donors were guided by a native shrewdness and common sense that kept them from the blunders of their descendants. They left sums to be expended in apprenticing boys, in training girls in domestic work, in educating children. These benefactions have in many instances been deflected from their original direction, with more or less wisdom; and doubtless in the administration of many there has been abuse; but the original idea is unmistakeably sound, namely, to put the beneficiaries in the way of becoming self-supporting; the gift of technical training or education to be a temporary thing, a loan to be repaid, though indirectly, to the community at large, by increased efficiency in labour when manhood should be reached. The old donor never intended that the boy who was educated through his charity should do so little for his own children that they in turn should require to be maintained out of the same or a similar fund. Such an improvident person our donor would have been the first to condemn as a rogue and vagabond, who would assuredly be deported beyond the bounds of the parish, not without some stripes, and exceeding fortunate if he carried his ears still comfortably attached to his head. And in his instincts, however harsh his methods may seem to our super-sensitive generation, the old donor was right. The philanthropy that does not elevate tends to pauperise, and the field of philanthropy is not to be occupied by what M. Louis Paulian, in his "Paris qui mendie," wittily calls *paupericulture*.

It would be too much to say, however, that no form of charity is justified which does not bear the promise of being repaid at some future time. That sentiment of humanity which forbids us to see a fellow-creature die from want of the necessaries of life justifies our making provision for the blind, the deaf, the insane, and the aged. Modern intelligence, however, avails to enable the two first to earn their own bread, at least in considerable measure. When a blind man begs in the public street we may be sure that it is with the well-founded assurance that he will thus make a better living than he would at any craft to which he could be put. The insane must be kept; but it is only right that where it is possible their relations should contribute to their support. It is rather strange, and shows how little reasoning has to do with the impulses of so-called charity, that it was only in 1828 that there was any State provision made for pauper lunatics, while out-relief to the able-bodied poor, in aid of insufficient wages, had been granted in 1796. Of this last form of charity we shall speak later on, but at present it is enough to note, as a proof that emotion is a poor guide in matters of philanthropy, that no provision was made for the most helpless and unfortunate class of people, while public money was lavished on those who ought to have been able to earn their own living.

The claim of the aged to support and shelter in their declining years, although admissible, is less strong. Cases no doubt arise where parents, having spent the earnings of their vigorous days in the bringing up of their children, see these children die before them, and are left helpless and destitute in their old age. But such cases are rare. Very few parents but have children whose direct duty it is to help them in their old age. Where there is a family of any size, such maintenance as the old people need can be given without too great a sacrifice; there are, indeed, but few cases where it will not be given if it is a choice between keeping the parents and letting them go to the workhouse; but out-relief is, unfortunately, regarded simply as a convenient addition to the family income, which may be taken without shame or humiliation. Most single persons also have relations, who would support them if neither the State nor a charitable institution were ready to do so; and it must be remembered that in a great number of cases single men and women have had the power, had they but had the will and the self-control necessary, to make some provision for their old age. With women of the middle and upper classes this was until lately by no means commonly the case. English parents have not the wise French custom of making some provision for their daughters; mistaken notions of gentility prevented them from letting their daughters go out to work, and thus we had the pitiful spectacle of refined and sensitive women reduced to starvation or beggary, and in many cases preferring the former. We may hope that "Indigent

* "Political Economy." By Francis A. Walker, Professor of Political Economy and History in the Sheffield Scientific School of Yale College. Page 367.

Gentlewomen's Societies" will have less work in the future than they have had in the past, but we fear that the time will never come when private charity will not find some such objects on which to expend itself. But at least these women, as they often say with tears in their eyes, never expected to need charity, and in their case the acceptance of it is not so demoralising as it is where the giving of charity to one teaches others to demand it. It may be harsh to say that the man or woman who expects to be supported by charity in his old age should for that very reason be refused it; but the fact remains that the knowledge that he will not be allowed to die of hunger makes many a man who might save for the future, by denying himself indulgences of a useless even if not of a vicious nature, live entirely from hand to mouth.

